# A Stage-Dependent Translational Signature in Peripheral Blood Mononuclear Cells from Clinically Isolated Syndrome to Relapsing-Remitting Multiple Sclerosis: An Exploratory Cross-Sectional Study

**DOI:** 10.3390/ijms27167340

**Published:** 2026-08-17

**Authors:** Simone D’Angiolini, Aurelio Minuti

**Affiliations:** IRCCS Centro Neurolesi “Bonino-Pulejo”, Via Provinciale Palermo, Contrada Casazza, 98124 Messina, Italy

**Keywords:** multiple sclerosis, clinically isolated syndrome, relapsing-remitting multiple sclerosis, PBMCs, ribosome, translation, transcriptomics

## Abstract

Multiple sclerosis (MS) is a chronic autoimmune disease of the central nervous system characterized by inflammatory demyelination, neurodegeneration, and progressive disability. Clinically isolated syndrome (CIS) often represents the first overt presentation of MS. We performed an exploratory cross-sectional transcriptomic investigation of peripheral blood mononuclear cells (PBMCs) from healthy controls (HC, *n* = 40), CIS patients (*n* = 49), and Relapsing-Remitting MS (RRMS, *n* = 53) patients using the ArrayExpress dataset E-MTAB-11415. Differential expression analysis was performed using limma, adjusting for age and sex. Functional enrichment and network analyses were conducted using clusterProfiler, STRING, and Cytoscape. Although Principal Component Analysis (PCA) showed partial overlap among groups, pathway-level analyses revealed coherent alterations in translation, ribosome biology, mitochondrial protein synthesis, and stress-response regulation. In CIS compared with HC, cytoplasmic and mitochondrial ribosomal genes were predominantly downregulated, suggesting reduced translational capacity in PBMCs. This was accompanied by altered expression of translation-initiation and transcriptional regulators, whereas genes involved in stress-adaptive translational control, including *GCN1*, *YARS1*, *QARS1*, and *SIL1*, were selectively upregulated. Conversely, RRMS compared with CIS showed upregulation of ribosome-related and biosynthetic programs. Overall, these findings suggest that CIS may be characterized by peripheral translational restraint and adaptive stress response activation, whereas RRMS progression is associated with biosynthetic reactivation.

## 1. Introduction

Multiple sclerosis (MS) is a chronic autoimmune disease of the central nervous system (CNS) characterized by inflammatory demyelination, neuroaxonal injury, and progressive neurodegeneration, which together contribute to variable degrees of neurological disability [1]. Although MS is relatively uncommon during childhood, its incidence rises after adolescence, with peak prevalence occurring between 20 and 40 years of age and a mean age at onset of approximately 30 years. Moreover, women tend to develop symptoms approximately 2–5 years earlier than men [2]. In Europe, MS has an estimated prevalence of approximately 83 cases per 100,000 individuals, with an average annual incidence of 4.3 new cases per 100,000 individuals [3].

Despite major advances in the understanding of MS pathophysiology, the mechanisms initiating and sustaining the autoimmune response remain only partially understood. In the early stages of the inflammatory cascade, immune responses are thought to target myelin-associated antigens, including myelin basic protein, proteolipid protein, myelin oligodendrocyte glycoprotein, and gangliosides. At the neuropathological level, MS lesions are commonly classified as acute, chronically active, or inactive, highlighting the spatial and temporal heterogeneity of inflammatory demyelination within the CNS [4].

The earliest clinical manifestation of MS is frequently represented by clinically isolated syndrome (CIS), defined as a first episode of acute or subacute neurological dysfunction caused by inflammatory demyelination involving the optic nerve, brainstem, spinal cord, or other CNS regions. CIS typically affects young adults, reaches maximal clinical severity within 2–3 weeks, and represents the initial presentation of MS in approximately 85% of cases [5]. For an episode to be classified as CIS, symptoms must persist for at least 24 h, occur in the absence of fever or infection, and present without signs of encephalopathy [6,7].

Assessing the risk of conversion from CIS to clinically definite MS is clinically relevant because early conversion is associated with worse prognosis and greater long-term disability. Patients considered at higher risk may therefore benefit from earlier or more effective disease-modifying therapies, whereas lower-risk patients may be managed with safer therapeutic strategies [8]. However, the clinical course after CIS remains highly heterogeneous. After 15–20 years, approximately one-third of patients show a relatively mild disease course with little or no disability, whereas nearly half develop secondary progressive MS, characterized by gradual and irreversible disability accumulation [6].

Current predictors of conversion from CIS to MS include the presence of asymptomatic Magnetic Resonance Imaging (MRI) lesions and oligoclonal bands (OCBs) in the cerebrospinal fluid (CSF). By contrast, factors such as vitamin D deficiency, Epstein–Barr virus exposure, smoking, Human Leukocyte Antigen-associated genetic susceptibility, and immunological abnormalities are considered relevant but less consistent predictors [6]. According to the McDonald diagnostic criteria, a patient presenting with CIS can be diagnosed with MS when MRI evidence demonstrates dissemination in space and dissemination in time, or when CSF OCBs substitute for dissemination in time in the appropriate clinical context [9].

Although MRI and CSF parameters remain essential in clinical practice, they do not fully capture the molecular heterogeneity of CIS or the systemic immune mechanisms that may precede conversion to clinically definite MS. In this context, peripheral blood represents a clinically accessible compartment for biomarker discovery. Blood-based biomarkers may provide relevant information on disease-related immune processes, treatment response, and systemic biological changes associated with MS [10]. Moreover, the blood transcriptome reflects molecular signals originating from multiple tissues and immune cell populations, thereby providing an integrated snapshot of systemic biological activity [11].

Peripheral blood mononuclear cells (PBMCs) are particularly relevant for studying MS because they include immune cell populations directly involved in disease pathogenesis, including T cells, B cells, monocytes, and natural killer cells. While previous transcriptomic studies have largely focused on inflammatory mediators, antigen presentation, cytokine signaling, and lymphocyte activation, the contribution of ribosomal activity, translational control, mitochondrial protein synthesis, and stress-response pathways remains less explored. These processes are critical for immune cell activation, metabolic adaptation, cytokine production, proliferation, and effector function.

In the present study, we analyzed transcriptomic profiles from peripheral blood samples of HC, CIS patients, and Relapsing-Remitting Multiple Sclerosis (RRMS) patients to characterize disease-stage-associated molecular signatures in the peripheral immune compartment. Particular attention was given to transcriptional changes involving ribosomal and mitoribosomal genes, translational regulation, and stress-response pathways. By defining these alterations, this study aims to provide new insights into early peripheral immune remodeling in CIS and to identify candidate molecular signatures potentially associated with progression toward clinically definite MS. To address these questions, we conducted an exploratory cross-sectional transcriptomic analysis of PBMCs using publicly available microarray data (E-MTAB-11415). This study aims to map stage-specific transcriptomic dynamics across the early MS spectrum.

## 2. Results

**Figure 1:**
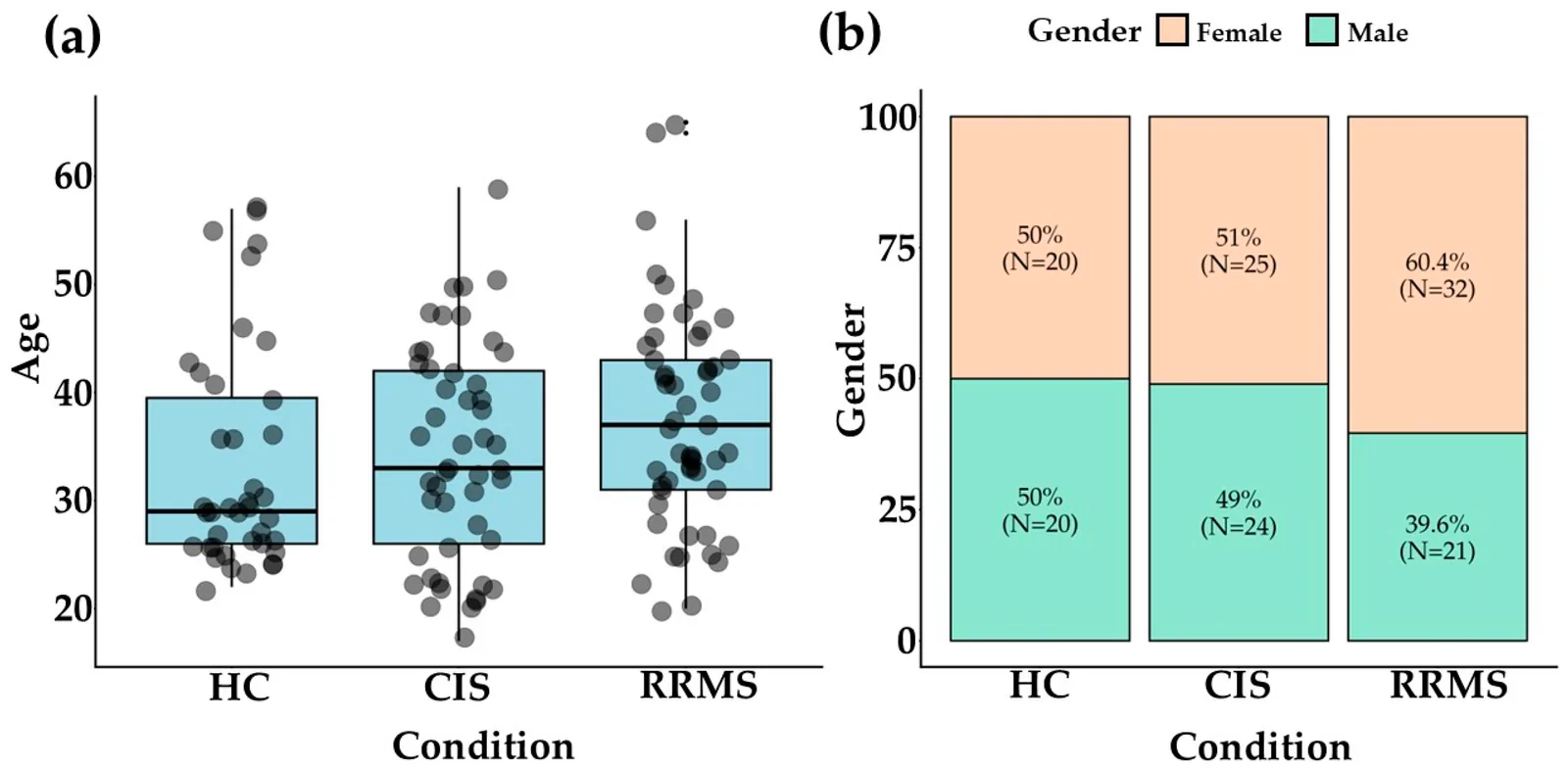
Demographic and clinical characteristics of the study cohort. (**a**) Age distribution across healthy controls (HCs), CIS and RRMS patients. (**b**) Sex distribution, expressed as percentage and count of male and female participants, within each clinical group.

In accordance with the inclusion criteria described in the Materials and Methods Section, the dataset E-MTAB-11415 was selected from the ArrayExpress repository for this study. The cohort comprises samples from HC, as well as patients diagnosed with CIS and RRMS. Selection was contingent upon the availability of comprehensive metadata for each sample, specifically clinical status, age, and biological sex, to ensure the statistical validity of the subsequent analysis. The demographic and clinical profile of the study cohort is illustrated in Figure 1. Specifically, Figure 1a outlines the age distribution across the three clinical groups, highlighting the progressive increase in median age aligned with the disease spectrum, while Figure 1b details the precise percentage and count of male and female participants within each condition.

Samples in this dataset represent a single peripheral blood draw per subject, obtained at one time point per individual; no subject was sampled longitudinally or at multiple disease time points. Consequently, HC, CIS, and RRMS groups correspond to independent, cross-sectionally sampled cohorts rather than to serial samples from the same patients followed over time. The publicly available metadata for E-MTAB-11415 do not specify the timing of sample collection relative to the index demyelinating event for CIS patients, nor clinical follow-up data on subsequent conversion to definite MS; this constraint is discussed further in Section 3.6.

**Figure 2:**
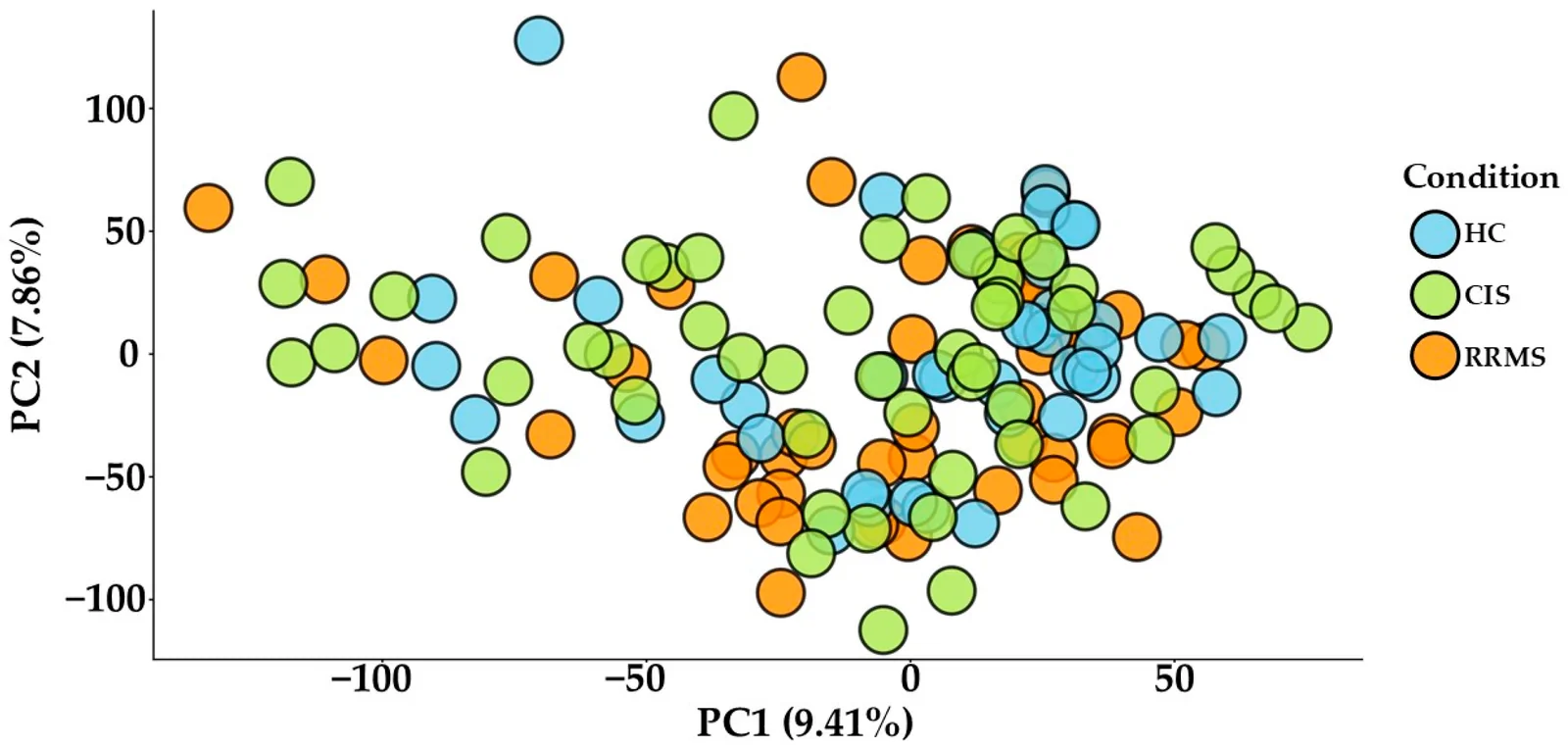
Principal Component Analysis (PCA) of transcriptomic profiles across clinical conditions. PCA based on normalized, log_2_-transformed expression intensities, demonstrating the distribution and clustering of biological replicates across three experimental groups: healthy controls (HCs, light blue), Clinically isolated syndrome (CIS, light green), and Relapsing-Remitting Multiple Sclerosis (RRMS, orange). The first principal component (PC1) and second principal component (PC2) explain 9.41% and 7.86% of the total variance, respectively, illustrating the global expression landscape and phenotypic overlap among the studied clinical cohorts.

To evaluate the global transcriptomic variance and assess the overall distribution of the samples within the E-MTAB-11415 dataset, a Principal Component Analysis (PCA) was performed on the normalized expression data. This exploratory step was essential to determine whether the global gene expression profiles could naturally distinguish among the three clinical cohorts: HC, CIS, and RRMS, prior to downstream differential expression workflows. The PCA scores plot, defined by the first two principal components, captured a cumulative variance of 17.27%, with PC1 and PC2 accounting for 9.41% and 7.86% of the total transcriptomic variance, respectively (Figure 2). As illustrated in Figure 2, the spatial distribution of the samples revealed a substantial overlapping pattern among the HC, CIS, and RRMS groups across both axes, indicating that the baseline transcriptomic profiles share a high degree of global similarity without clear-cut, unsupervised macro-clusters separating the conditions. This lack of distinct separation highlights the complexity of the circulating transcriptomic signals and confirms the absence of dominant, confounding technical batch effects dominating the dataset structure. Consequently, this multi-dimensional distribution justifies and establishes a solid foundation for conducting subsequent, more sensitive pairwise differential expression analyses to isolate specific, statistically significant alterations associated with each clinical condition.

**Figure 3:**
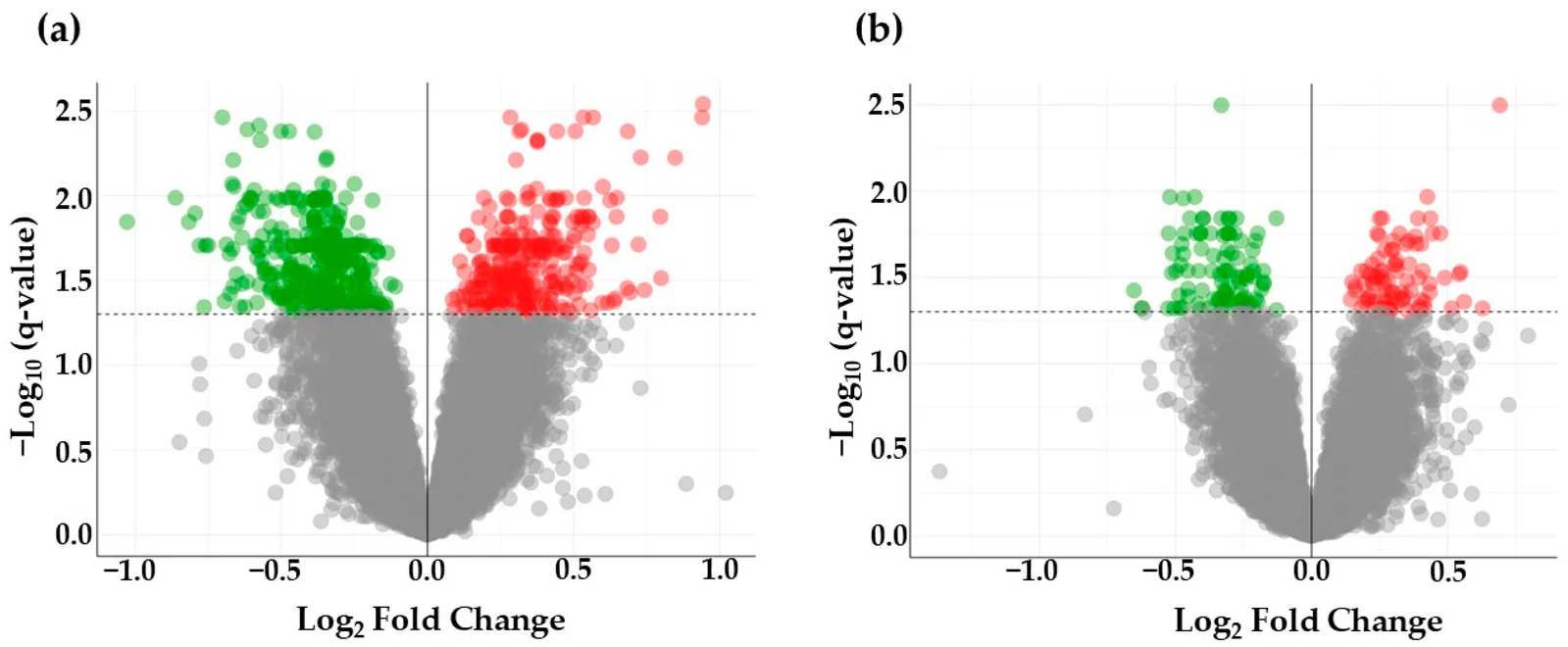
Volcano plots show all the probes inspected in the different comparisons. The first plot (**a**) refers to the comparison HC vs. CIS, while the second plot (**b**) refers to the comparison CIS vs. RRMS. In both plots, we report the Log_2_ Fold Change on the x-axis and the −log_10_ q-value on the y-axis with a horizontal line that marks the statistical threshold set at 0.05. Grey dots represent non-significant genes, green dots represent significantly downregulated genes, and red dots represent significantly upregulated genes.

To isolate specific, statistically significant alterations associated with disease onset and subsequent clinical progression, pairwise differential expression analyses were executed using linear models adjusted for age and biological sex covariates. Transcriptomic changes were analyzed sequentially across two major clinical transitions: HC vs. CIS (disease initiation) and CIS vs. RRMS (disease progression). The differential expression framework was implemented using linear models with empirical Bayes moderation (limma). To control the false discovery rate and minimize the accumulation of false-positive Differentially Expressed Genes (DEGs), the raw *p*-values generated by the linear modeling were rigorously adjusted using the Benjamini–Hochberg (BH) post hoc correction method. Transcripts demonstrating an adjusted significance level, expressed as a q-value, below the threshold of 0.05 (q-value < 0.05) were considered statistically significant DEGs and selected for subsequent downstream workflows. The overall distribution of the magnitude and statistical significance (−log_10_ q-value) of these structural transcription fluctuations was visualized via Volcano plots for both distinct experimental comparisons (Figure 3a,b).

The analysis yielded a distinct number of DEGs for each pairwise comparison based on the selected statistical criteria. Specifically, the contrast monitoring disease initiation (HC vs. CIS) revealed a total of 672 statistically significant DEGs were identified. Within this baseline comparison, the transcripts were further stratified based on their regulation profile: 294 genes were identified as upregulated, while 378 genes were classified as downregulated. In this context, downregulated genes signify that their transcriptional expression levels are significantly decreased in patients with CIS compared to the HC baseline. Conversely, upregulated genes characterize transcripts whose expression levels are significantly increased in the CIS cohort relative to the HC group. In contrast, the comparison evaluating disease progression from the early clinically isolated stage to the relapsing-remitting phenotype (CIS vs. RRMS) identified a smaller, more refined transcriptomic signature comprising 187 significant DEGs. Within this progression cohort, 88 genes were identified as upregulated, while 99 genes were classified as downregulated. Here, downregulated genes imply that their transcriptional expression levels are significantly decreased in the RRMS phenotype compared to the CIS baseline stage, whereas upregulated genes characterize transcripts whose expression levels are significantly increased in patients who have transitioned to the RRMS stage relative to the CIS cohort.

**Figure 4:**
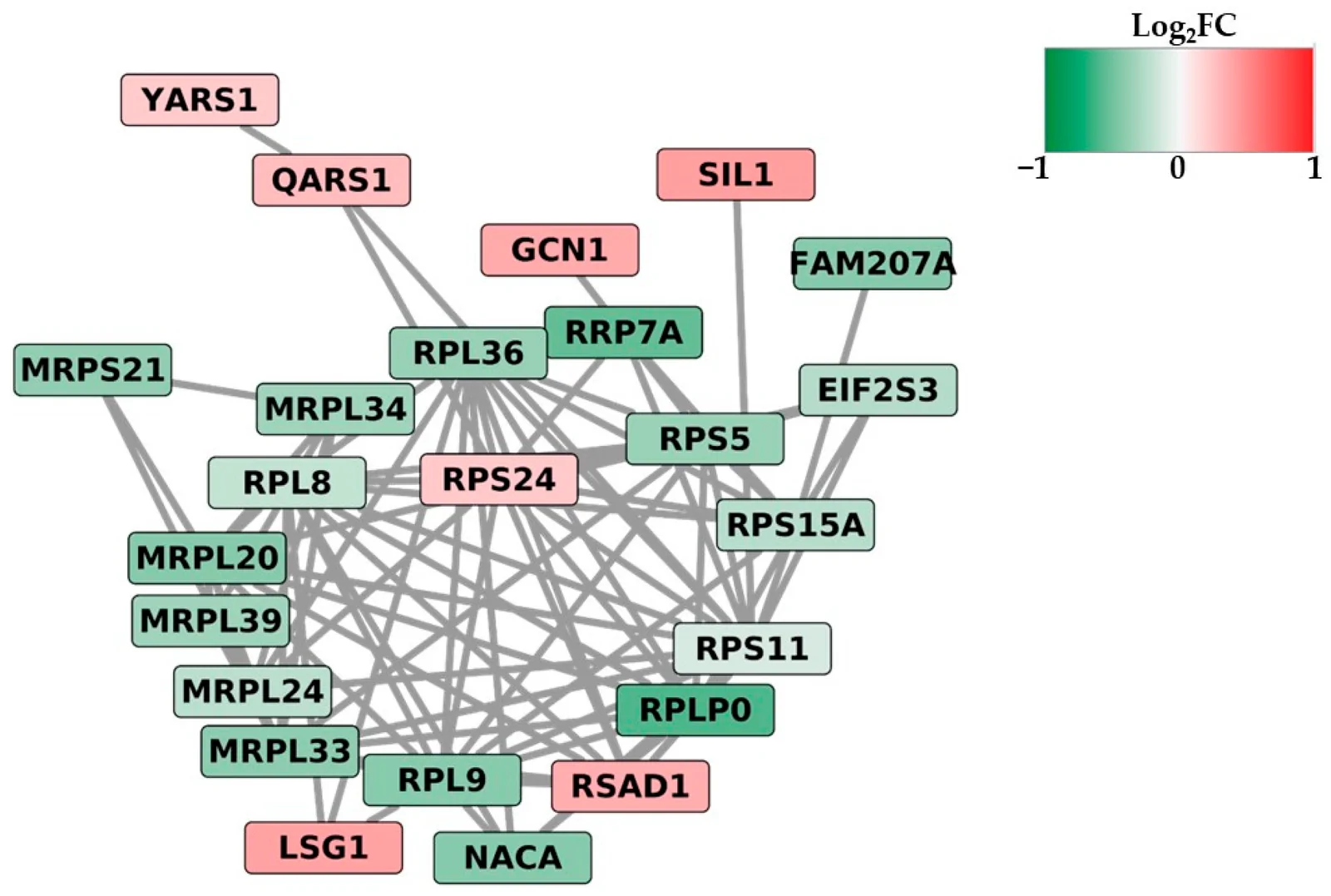
Topological analysis of the core protein–protein interaction (PPI) subnetwork. Functional interaction architecture of the core subnetwork isolated from the global interactome evidence. Node color transitions from green to red according to the log_2_ Fold Change scale, indicating downregulated and upregulated transcripts, respectively.

It is worth noting that no statistical threshold was applied to the Fold Change (FC) values during the selection of the DEGs. This deliberate strategy was adopted to comprehensively evaluate the global transcriptomic architecture. Indeed, subtle expression fluctuations can exert a profound physiological impact when acting coordinately within complex biological systems. Furthermore, imposing a stringent FC filter would have inherently compromised the integrity of the subsequent topological network analysis, risking the exclusion of critical biological hubs and intermediate nodes that are essential to fully map the systemic connectivity of the altered pathway. To explore the functional interaction architecture of these DEGs, the complete list of altered transcripts was mapped using the STRING database. Protein–protein interactions (PPI) were filtered using a high-confidence threshold (required score > 0.700), and network noise was minimized by strictly excluding interactions based exclusively on text-mining evidence. Through this topological approach, a global protein interaction network comprising 118 nodes connected by 229 edges was successfully constructed (fully consultable in Supplementary Figure S1). From this global interactome, a core functional subnetwork capturing the genes with the highest degree of connectivity was systematically isolated and visualized (Figure 4).

**Figure 5:**
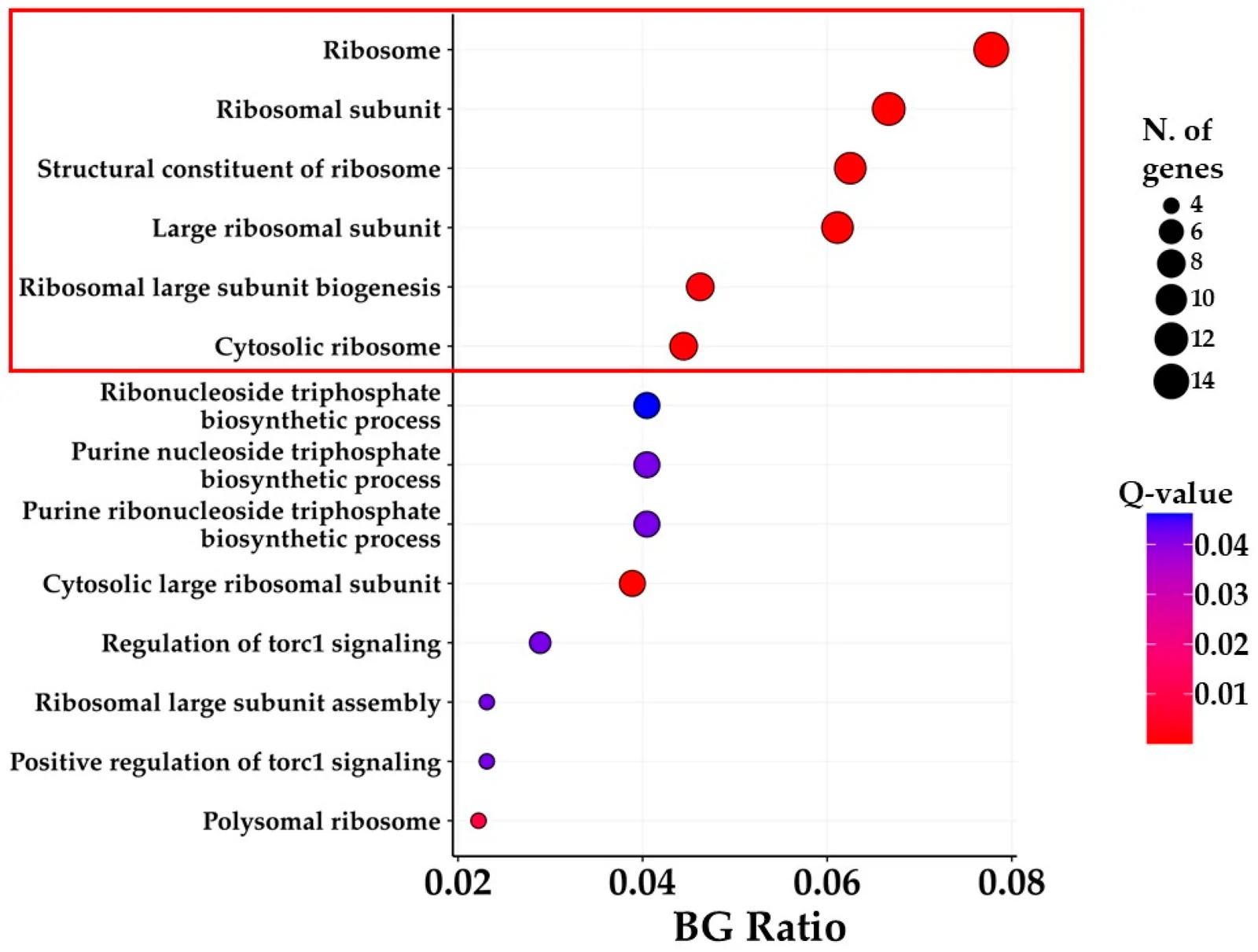
Over-Representation Analysis (ORA) of Gene Ontology (GO) terms in the CIS vs. RRMS contrast. Dot plot visualization of the enriched GO categories (Biological Processes, Cellular Components, and Molecular Functions) DEGs CIS vs. RRMS comparison. The *x*-axis represents the Background Ratio (BG Ratio). The size of each dot corresponds to the absolute number of genes associated with the respective term (N. of genes), while the color gradient indicates the statistical significance based on the Benjamini–Hochberg adjusted *p*-value (Q-value), spanning from red (highest significance, q-value 0.01) to blue (q-value 0.05).

As illustrated in Figure 4, the highlighted ribosomal subnetwork demonstrated a clear, generalized trend toward translational suppression, characterized by the marked downregulation (represented in green) of essential structural components and assembly factors. The complete list of these transcripts along with their corresponding FC values is detailed in Supplementary Table S1. Following these findings, we investigated whether the evidence of this ribosomal machinery alteration was also sustained during the subsequent stage of disease progression. Therefore, we performed an Over-Representation Analysis (ORA) of Gene Ontology (GO), specifically on the DEGs isolated from the CIS vs. RRMS comparison. Results of the ORA analysis are graphically summarized in Figure 5.

**Figure 6:**
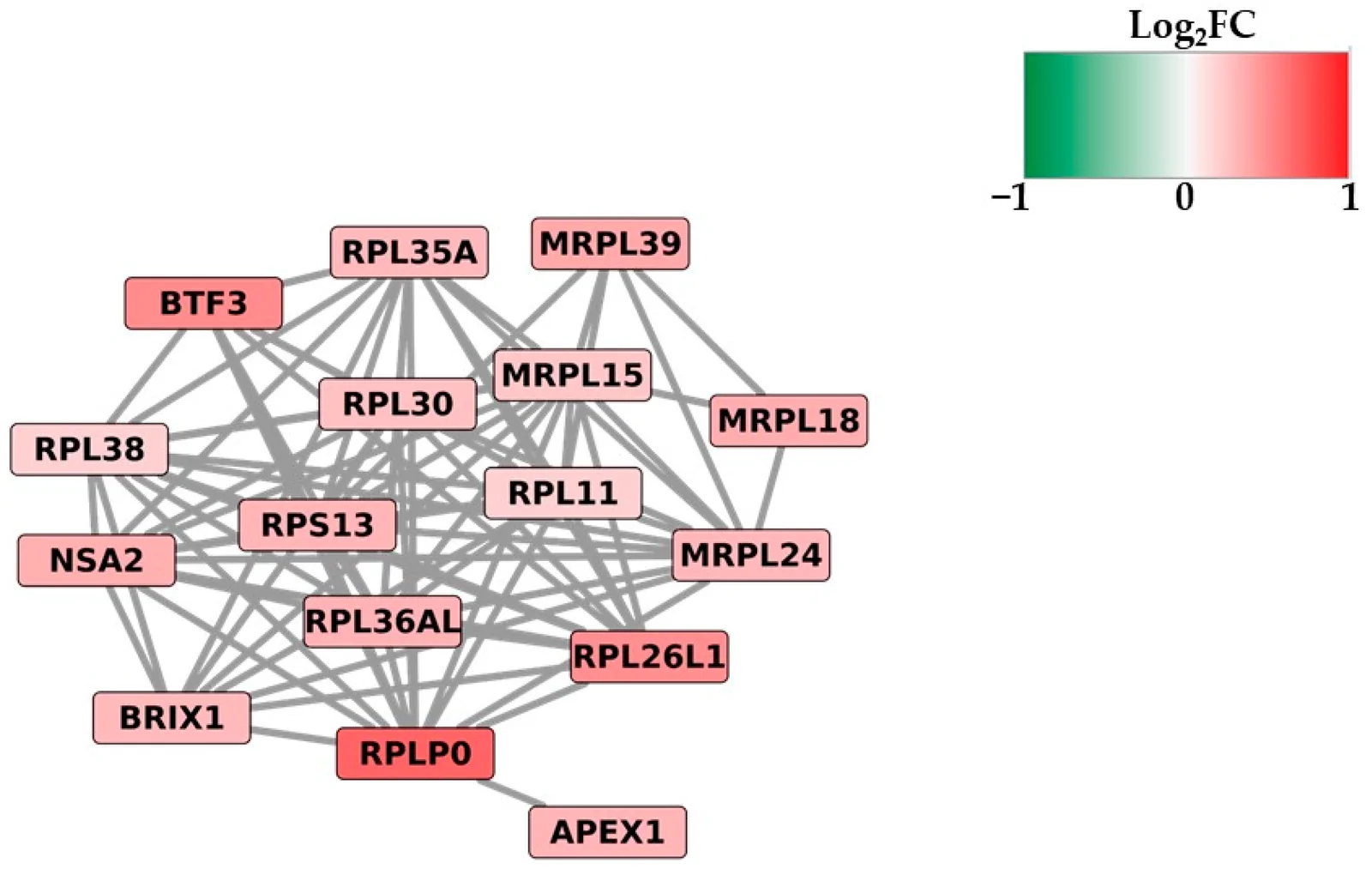
Ribosomal network in CIS vs. RRMS. Nodes are color-coded based on their Log_2_ Fold Change value, showing a homogeneous and generalized trend toward upregulation (red gradient) in this specific disease transition.

Visual inspection of the resulting dotplot revealed a stark predominance of ribosome-related terms among the top-ranked functional categories. Specifically, the six ontologies exhibiting the highest gene counts spanned all three GO domains: Cellular Component (CC: GO:0005840~ribosome, CC: GO:0044391~ribosomal subunit, CC: GO:0015934~large ribosomal subunit, and CC: GO:0022626~cytosolic ribosome), Molecular Function (MF: GO:0003735~structural constituent of ribosome), and Biological Process (BP: GO:0042273~ribosomal large subunit biogenesis). Given the intrinsic hierarchical structure of GO and the functional overlap between these highly enriched cascades, we observed a substantial redundancy in their gene composition. To streamline the downstream analysis and eliminate redundant annotations, the gene sets corresponding to these six leading ontologies were merged. This consolidation strategy yielded a non-redundant core set of 17 unique genes. To further elucidate the molecular interplay and coordinated regulation among these core components, the 17 merged genes were uploaded to STRING, resulting in a network of 16 nodes. The structural architecture and connectivity patterns of this centralized ribosomal network are illustrated in Figure 6.

**Figure 7:**
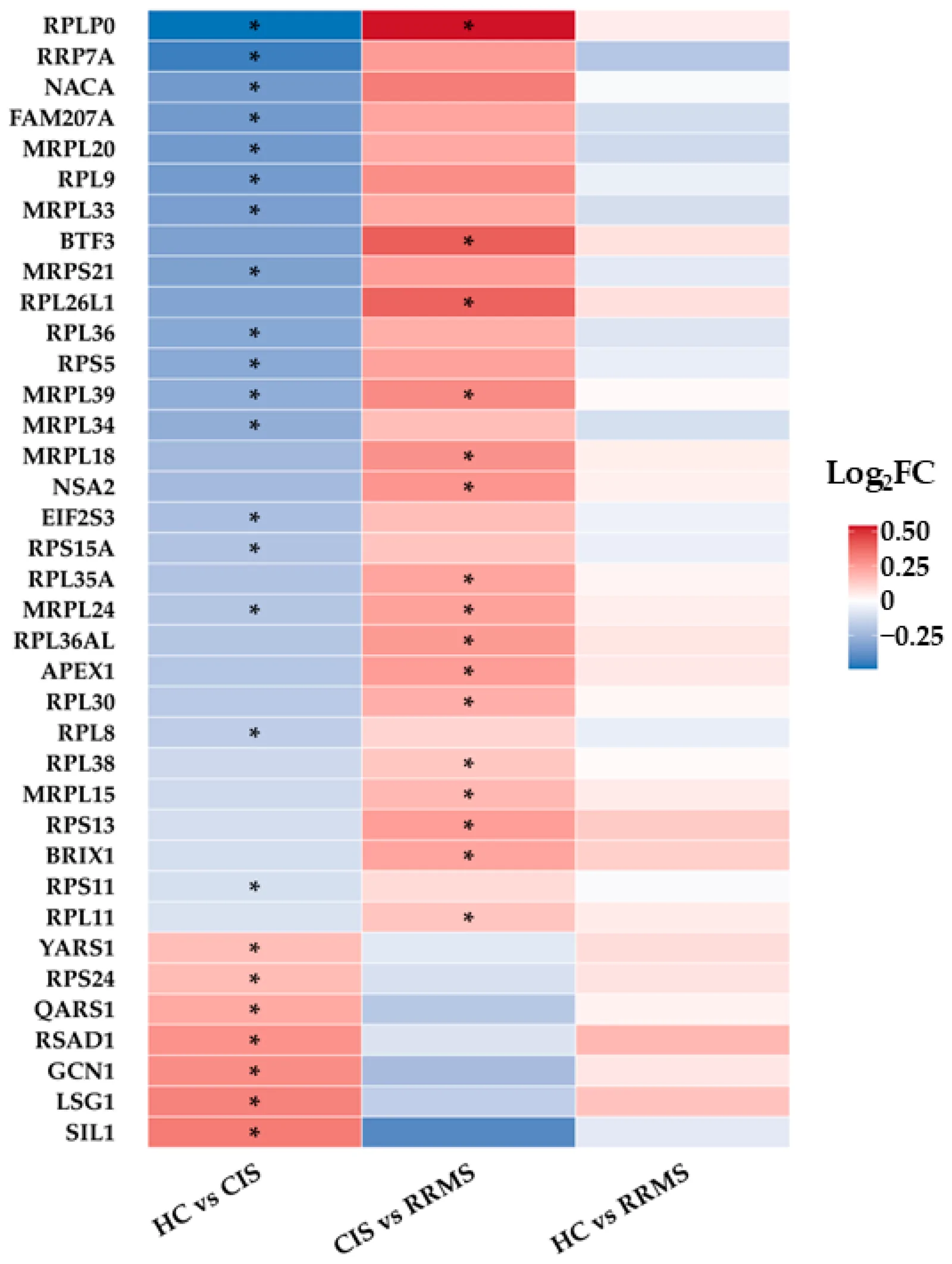
Heatmap of log_2_ Fold Change for the 37-gene panel drawn from the HC vs. CIS protein–protein interaction network (Figure 4) and the CIS vs. RRMS ribosomal/mitoribosomal core network (Figure 6), shown across the HC vs. CIS, CIS vs. RRMS, and HC vs. RRMS comparisons. Asterisks indicate statistical significance (* q < 0.05, Benjamini–Hochberg adjusted). Genes are ordered by expression pattern across the three contrasts.

While the CIS phase displayed a mixed expression profile, a closer inspection of the corresponding network in the RRMS phase (Figure 6) reveals that all 17 genes are systematically and uniformly upregulated. This highly coordinated behavior demonstrates a clear divergence in ribosomal gene expression between the CIS and the RRMS stages. The simultaneous activation of structural components belonging to both the cytosolic ribosome (e.g., *RPLP0, RPL35A, RPS13*) and the mitochondrial ribosomal machinery (*MRPL15, MRPL39*) strongly implies an accelerated translational demand and protein synthesis reactivation. This evident transcriptional divergence underscores the dynamic rewiring of translational regulation as a prominent molecular hallmark differentiating these two clinical phases. To determine whether the transcriptional differences identified in the HC vs. CIS and CIS vs. RRMS comparisons persist into established RRMS, we examined a direct contrast between RRMS and HC. An apparent shift toward HC-comparable levels between CIS and RRMS does not necessarily imply that expression fully normalizes: the magnitude of this shift could be incomplete, leaving genes still measurably different from HC, or could overshoot the original baseline, resulting in a divergence in the opposite direction. To test this directly, we compiled a panel of 37 genes drawn from the HC vs. CIS protein–protein interaction network (Figure 4, *n* = 21) and the CIS vs. RRMS ribosomal/mitoribosomal core network (Figure 6, *n* = 13), with three genes (*RPLP0, MRPL24, MRPL39*) common to both, and examined their expression directly in the RRMS vs. HC comparison. Results of this analysis are summarized in Figure 7.

Full log_2_ fold-change, *p*-value, and q-value data for each gene across all three comparisons, along with their functional category assignment, are reported in Supplementary Table S2.

**Figure 8:**
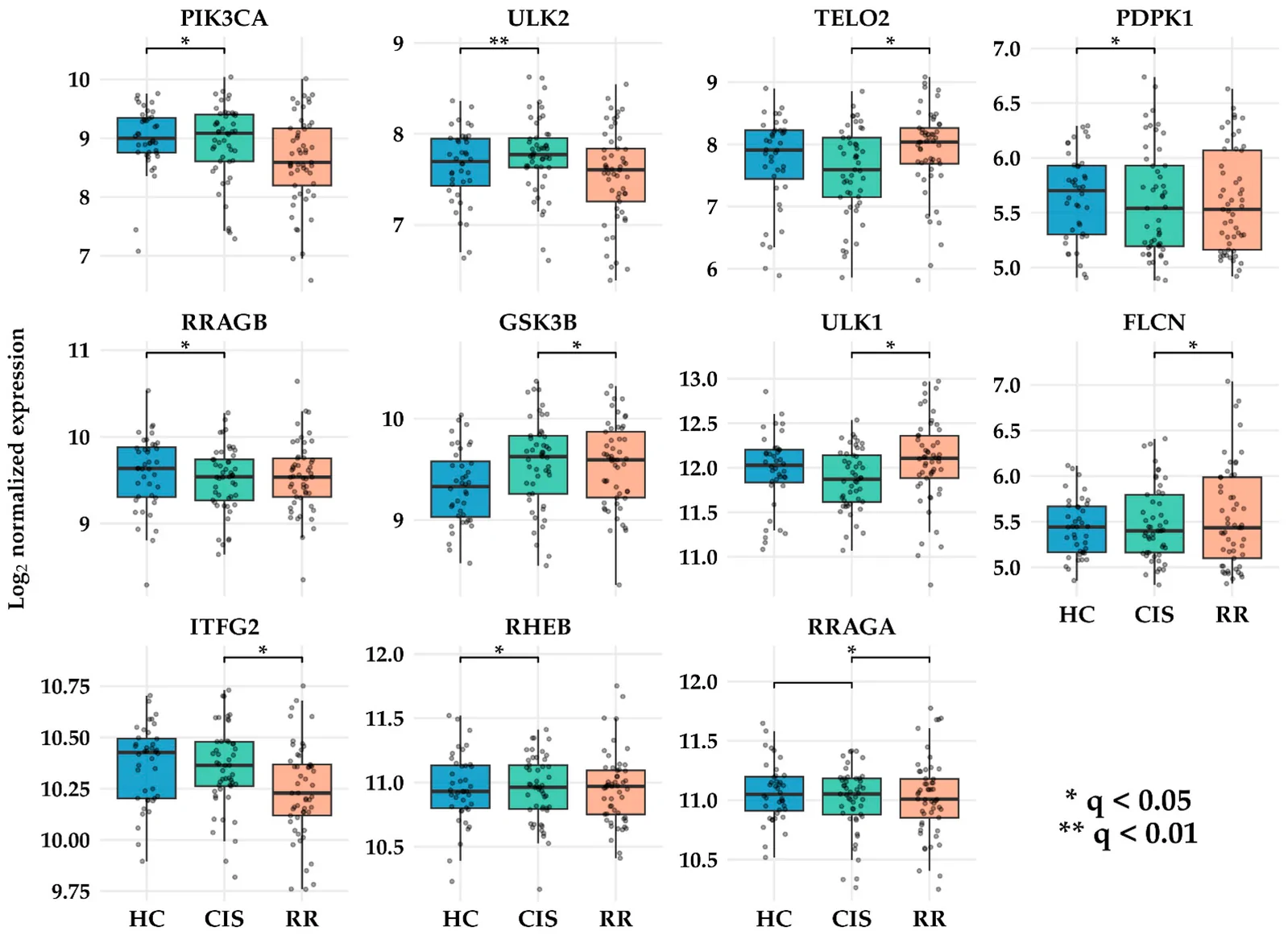
Normalized expression of mTORC1 pathway genes significantly altered in HC vs. CIS and/or CIS vs. RRMS. Boxplots show log_2_-normalized expression across HC, CIS and RRMS groups for the 11 genes of the curated 19-gene mTORC1 panel (Supplementary Table S3), reaching q < 0.05 in at least one of the two comparisons; individual data points are overlaid as jittered dots. Horizontal brackets and asterisks indicate the specific comparison(s) in which each gene reached statistical significance (* q < 0.05, ** q < 0.01, Benjamini–Hochberg adjusted).

Given that mTORC1 signaling and the unfolded protein response (UPR) are established upstream regulators of ribosome biogenesis and cap-dependent translation, the same translational program found to differ in direction between CIS and RRMS above, and given their documented role in oligodendrocyte precursor cell (OPC) differentiation and myelination, we asked whether either pathway showed a parallel, stage-associated shift. We assembled literature-curated panels of core pathway components (mTORC1: amino-acid sensing via the Rag-Ragulator-FLCN-TTT complex (*RRAGA/B/C/D, FLCN, TELO2, ITFG2*), the upstream PI3K-AKT-TSC-RHEB axis (*PIK3CA, PDPK1, AKT1, TSC1, TSC2, RHE*), and downstream effectors/autophagy initiation (*ULK1, ULK2, GSK3B, EIF4EBP1, RPS6KB1, EIF4E*); UPR: ER chaperones/folding machinery (*HSPA5, HSP90B1, DNAJB9, DNAJC3, CALR, CANX, PDIA3/4/6, HYOU1, SDF2L1*) and the three canonical branches, PERK (*EIF2AK3, EIF2S1, ATF4, DDIT3, PPP1R15A*), IRE1 (*ERN1, XBP1*) and ATF6/ERAD (*ATF6, EDEM1, HERPUD1, SEL1L, SEC61A1*) and examined their expression across the HC vs. CIS and CIS vs. RRMS comparisons (Supplementary Tables S3 and S4). One UPR gene, *ATF4* (PERK branch), reached significance, specifically in the HC vs. CIS comparison; no other UPR gene reached significance in either comparison (1/23). In contrast, several mTORC1 pathway components showed significant, largely reciprocal changes across the two stages (Figure 8). Eleven of the 19 panel genes reached significance in at least one comparison (Figure 8). Eight genes (*RRAGB, FLCN, TELO2, PIK3CA, PDPK1, ULK1, ULK2, GSK3B*) were upregulated in CIS relative to HC and downregulated in RRMS relative to CIS, while three genes (*RRAGA, ITFG2, RHEB*) showed the opposite pattern. Statistical significance, however, was largely comparison-specific rather than shared: five genes (*RRAGB, PIK3CA, PDPK1, RHEB, ULK2*) reached significance specifically in the HC vs. CIS comparison, five (*FLCN, TELO2, ITFG2, ULK1, GSK3B*) specifically in the CIS vs. RRMS comparison, and only one (*RRAGA*) in both.

This largely non-overlapping distribution suggests that mTORC1 pathway components may be modulated in a stage-restricted rather than uniformly progressive manner, a pattern broadly consistent with the stage-associated remodeling described for the ribosomal/translational signature, although the functional significance of this parallel remains to be established.

## 3. Discussion

In the present study, we analyzed the transcriptomic profile of PBMCs from HC, patients with CIS, and patients with RRMS to identify disease-stage-associated molecular signatures. By integrating differential expression analysis, functional enrichment, and network-based inference, we identified coherent alterations involving ribosomal biology, mitochondrial protein synthesis, transcriptional regulation, and cellular stress responses.

The main finding of this study is the identification of a stage-dependent remodeling of the translational machinery. CIS was characterized by the downregulation of several cytoplasmic and mitochondrial ribosomal genes, together with reduced expression of translation initiation and transcriptional components. In contrast, RRMS showed reactivation of ribosome-related and biosynthetic programs, suggesting a shift from an early stress-adapted state toward a more translationally active immune phenotype.

### 3.1. Translational Restraint and Stress Adaptation in CIS

The HC vs. CIS comparison revealed a coordinated downregulation of multiple ribosomal protein genes, including cytoplasmic components such as *RPL36*, *RPL8*, *RPL9*, *RPLP0*, *RPS5*, *RPS11*, and *RPS15A*, as well as mitochondrial ribosomal proteins such as *MRPS21*, *MRPL20*, *MRPL24*, *MRPL33*, *MRPL34*, and *MRPL39*. Since ribosomal proteins are required for ribosome assembly [12], translational fidelity [13], peptide elongation [14], and global protein synthesis [15], their reduction suggests decreased translational capacity in PBMCs from CIS patients. This finding is biologically relevant because immune cell activation requires substantial biosynthetic activity to sustain cytokine secretion, antigen-presentation machinery, receptor turnover, clonal expansion, and effector differentiation [16]. Therefore, the repression of ribosomal structural genes in CIS may reflect an early adaptive state in which peripheral immune cells limit global protein synthesis to reduce energetic demand and proteotoxic stress.

The concomitant downregulation of mitochondrial ribosomal genes adds an immunometabolic dimension to this signature. Mitoribosomes are required for the translation of mitochondrially encoded oxidative phosphorylation subunits; therefore, reduced expression of MRPS and MRPL family members may indicate altered mitochondrial protein synthesis and early immunometabolic remodeling [17,18,19]. In immune cells, mitochondrial function contributes not only to ATP production, but also to redox signaling, apoptotic threshold regulation, and cell fate decisions [20]. Thus, CIS PBMCs may undergo coordinated attenuation of both cytosolic and mitochondrial translation, potentially reflecting a metabolically constrained or stress-adapted immune cell state.

This interpretation is further supported by the downregulation of *EIF2S3*, a key component of the eukaryotic initiation factor 2 complex involved in translation initiation [21]. *EIF2S3* encodes the largest subunit of the heterotrimeric eukaryotic initiation factor 2 complex, which is required to deliver initiator methionine-tRNA to the small 40S ribosomal subunit during the early phase of translation initiation [22]. Since translation initiation is one of the major rate-limiting steps of protein synthesis, reduced *EIF2S3* expression strengthens the hypothesis that CIS PBMCs display suppression of canonical translational activity.

In parallel, downregulation of *POLR2E*, *POLR2J2*, *MED22*, *UXT*, and *NACA* genes suggests that this phenotype is not limited to ribosomal components, but may involve broader attenuation of the transcription-translation axis.

*POLR2E* encodes subunit E of RNA polymerases I, II, and III, representing an essential component of the cellular transcriptional apparatus involved in the production of both protein-coding and non-coding RNAs. As a common subunit shared by all three nuclear RNA polymerases, *POLR2E* contributes to DNA-dependent RNA synthesis and participates in the formation of the lower jaw domain of RNA polymerase II, which helps correctly position the DNA template within the catalytic cleft [23,24].

*POLR2J2* encodes RNA polymerase II subunit J2, a protein associated with the RNA polymerase II complex that mediates DNA-dependent transcription and mRNA production in eukaryotic cells [25].

*MED22* encodes subunit 22 of the Mediator complex, a transcriptional regulatory protein that helps connect gene-specific transcription factors with RNA polymerase II and the general transcription machinery. Through its role within the Mediator complex, MED22 contributes to RNA polymerase II-dependent transcription by integrating regulatory signals with the molecular apparatus responsible for initiating and sustaining gene expression. It is also implicated in the assembly of the RNA polymerase II preinitiation complex and in the positive regulation of transcription initiation and elongation. The encoded protein is found in the nucleus, nucleoplasm, and cytoplasm, consistent with its involvement in transcriptional control [26].

*UXT* encodes a prefoldin-like co-chaperone involved in the regulation of transcriptional activity and apoptotic processes, while also playing a role in the control of mitochondrial dynamics [27]. Emerging evidence also links UXT to neurodegeneration-related mechanisms, particularly ALS-associated proteotoxicity, where UXT promotes p62/SQSTM1-dependent aggrephagy and facilitates the clearance of mutant SOD1 aggregates, thereby delaying motor neuron degeneration in experimental models [27].

However, this suppression was not uniform. Several genes involved in stress-adaptive translational control were selectively upregulated, including *GCN1, YARS1*, *QARS1*, and *SIL1. GCN1* encodes a ribosome-associated regulator of translation that detects ribosome collisions occurring during translational arrest and contributes to activation of the integrated stress response. Its localization to cytosolic ribosomes and the cytosol is consistent with a role at the crossroads of translational quality control and cellular stress signaling [28]. GCN1 is functionally linked to amino acid stress sensing and activation of the GCN2/EIF2AK4 pathway in response to uncharged tRNAs [28]. Activation of this pathway leads to eIF2α-dependent translational control, reducing general protein synthesis while allowing preferential translation of stress-adaptive transcripts, including ATF4-regulated programs [29,30]. Therefore, increased *GCN1* expression in CIS may indicate that PBMCs engage a stress-sensing program aimed at adapting to metabolic, amino acid, oxidative, or inflammatory stress.

Similarly, increased expression of *YARS1* was observed, which encodes tyrosyl-tRNA synthetase 1, a class I aminoacyl-tRNA synthetase responsible for attaching tyrosine to its corresponding tRNA during protein translation [31]. In neurodegenerative disorders, YARS1 has been associated with peripheral neuropathies, such as Charcot–Marie–Tooth disease, as well as CNS conditions, including Alzheimer’s disease [32]. Similarly, *QARS1* encodes glutaminyl-tRNA synthetase 1, a class I aminoacyl-tRNA synthetase responsible for charging tRNA with glutamine during protein translation [33]. Its upregulation may reflect the preservation of tRNA aminoacylation and selective protein synthesis under stress conditions, whereas *SIL1* upregulation may reflect engagement of ER chaperone-assisted folding capacity. It should be noted that a broader, formally tested panel of UPR pathway genes did not show coordinated pathway-level engagement in either comparison (Section 3.4), so this SIL1-specific observation should not be interpreted as evidence of a canonical UPR response. Together, these findings support a model in which CIS PBMCs reduce broad housekeeping translation while maintaining selected adaptive pathways required for cellular survival, protein-folding homeostasis, and immune stress management.

### 3.2. RRMS Is Associated with Reactivation of Biosynthetic and Translational Programs

In contrast to CIS, RRMS was characterized by upregulation of ribosome-related genes and BP. This opposite pattern suggests a reactivation of translational and biosynthetic programs during the transition from CIS to RRMS. The increased expression of cytoplasmic ribosomal genes may reflect enhanced protein synthesis demand, supporting immune cell activation, proliferation, cytokine production, receptor remodeling, antigen processing, and effector functions.

The concomitant upregulation of mitochondrial ribosomal genes further suggests increased mitochondrial translational activity. Since mitoribosomes are required for the synthesis of mitochondrially encoded oxidative phosphorylation subunits, this profile may reflect enhanced energetic demand and mitochondrial remodeling in RRMS PBMCs.

Such a state would be consistent with the metabolic requirements of activated immune cells, which must coordinate protein synthesis, ATP production, redox signaling, and metabolic flexibility to sustain inflammatory activity. Overall, the opposite directionality observed between the HC vs. CIS and CIS vs. RRMS comparisons supports a disease-stage-dependent model of translational remodeling. In this model, CIS is marked by translational restraint and stress-adaptive regulation, whereas RRMS is associated with reactivation of ribosome biogenesis, mitochondrial translation, and biosynthetic capacity.

### 3.3. A Stage-Dependent Translational Model in Peripheral MS Immunity

Taken together, these findings suggest that CIS and RRMS may represent distinct peripheral immune states characterized by opposite regulation of the transcription–translation axis. In CIS, coordinated downregulation of cytoplasmic and mitochondrial ribosomal genes, together with reduced expression of translation initiation and transcriptional components, suggests a state of translational restraint. This profile is accompanied by selective activation of stress response and proteostasis-related genes, indicating that early demyelinating disease may involve adaptive repression of protein synthesis rather than generalized immune suppression.

In RRMS, the reactivation of ribosome-related and mitoribosomal programs may indicate a distinct, more biosynthetically active immune phenotype compared to CIS. This differential profile could support sustained immune responsiveness, inflammatory recurrence, and effector-cell activity. Therefore, the molecular differences observed between the CIS-like and RRMS-like profiles may reflect a shift from stress-adaptive translational repression toward biosynthetic reactivation across disease stages.

This stage-dependent translational model expands the interpretation of MS-related immune dysfunction beyond classical inflammatory pathways. It suggests that disease-stage-specific immune states may also be shaped by changes in ribosome biogenesis, mitochondrial translation, proteostasis, and metabolic adaptation. Importantly, the opposite directionality of ribosomal gene regulation between HC vs. CIS and CIS vs. RRMS supports the idea that these pathways are dynamically modulated across disease stages, rather than representing a nonspecific transcriptional alteration.

### 3.4. mTORC1 Signaling as a Candidate Upstream Node of the Stage-Dependent Translational Signature

The stage-dependent translational remodeling described above relative restraint in CIS, reactivation in RRMS prompted us to explore candidate upstream regulators that could plausibly contribute to such a pattern. mTORC1 is one of the principal integrators of nutrient, growth-factor, and energy-status signals and is known to influence ribosome biogenesis and cap-dependent translation, making it one candidate pathway potentially related to the ribosomal/mitoribosomal remodeling described in Section 3.1, Section 3.2 and Section 3.3. In line with this, several core mTORC1 pathway components showed significant, largely CIS-restricted changes that were not sustained into the CIS to RRMS transition (Figure 8), a pattern broadly reminiscent of the restraint-then-reactivation trajectory observed for the ribosomal signature, although the two sets of genes were only partially overlapping and the association should be regarded as descriptive rather than mechanistic.

Beyond its role in translational control, mTORC1 also has a described, cell-autonomous function in oligodendrocyte biology: conditional ablation of mTORC1 signaling (via Raptor) in oligodendrocyte lineage cells has been shown to impair oligodendrocyte differentiation and cause hypomyelination, particularly in the spinal cord [34,35], while oligodendrocyte-specific mTORC1 overactivation similarly disrupts myelination [34], indicating that balanced, rather than simply reduced or increased, mTORC1 activity is required for accurate CNS myelination [36]. Because our data derive from peripheral blood mononuclear cells rather than CNS-resident OPCs, they cannot establish any causal or cell-autonomous link to this process. Nonetheless, the CIS-restricted modulation of mTORC1 pathway components we observe is compatible with the more general possibility that peripheral changes in mTORC1-related signaling accompany the translational-restraint phenotype at disease onset, with a potential, currently untested, relevance to the myelination-permissive/non-permissive balance in early demyelinating disease. Given that individual gene changes were of mixed direction, this observation should be regarded as a pathway-level association rather than direct evidence of altered mTORC1 activity; functional validation (e.g., assessment of S6K1/4E-BP1 phosphorylation) would be needed to test this hypothesis directly. By contrast, we found no evidence of coordinated UPR engagement: only ATF4 (1/23 curated genes) reached significance, specifically in HC vs. CIS. As ATF4 is a downstream effector of the GCN2/eIF2α-driven integrated stress response already implicated by the GCN1 findings in Section 3.1, this isolated change likely reflects that same stress-adaptive axis rather than activation of the broader, ER-centered UPR program; taken together, these results argue against a major contribution of canonical ER-stress-driven mechanisms to this early signature.

### 3.5. Clinical Implications

Peripheral transcriptional signatures distinguishing CIS from RRMS may support disease stratification and progression risk assessment. Current predictors of conversion from CIS to clinically definite MS, such as MRI lesion burden and CSF OCB, are clinically useful but do not fully capture the molecular heterogeneity of early disease [6,9]. Peripheral blood transcriptomic markers may complement these tools by reflecting systemic immune activation, metabolic remodeling, and stress-response engagement [10,11].

The ribosomal/translational signature identified here may represent a candidate biomarker axis distinguishing early adaptive immune states from more established relapsing disease: a CIS profile dominated by ribosomal repression and stress-response activation vs. an RRMS profile marked by reactivation of ribosome biogenesis and biosynthetic capacity.

### 3.6. Limitations and Future Perspectives

This study has some limitations that should be considered. The design is cross-sectional, comparing independent cohorts rather than the same patients over time, so the observed differences reflect distinct molecular profiles associated with each stage rather than a documented conversion process. The clinical metadata available for E-MTAB-11415 are limited to age, sex, and clinical group, without disease duration, imaging, cerebrospinal fluid, or treatment information, which restricted our ability to adjust for these factors; the timing of sample collection relative to the index CIS event, as well as long-term clinical follow-up data on conversion to definite MS, are similarly not available, limiting our ability to relate the observed ribosomal/translational signature to a specific disease-onset window or to longer-term outcomes. Bulk PBMC profiling similarly does not resolve cell-subset composition, which may partly underlie the transcriptional differences reported. Future longitudinal cohorts integrating clinical, radiological, and immune cell-composition data, together with information on sampling time relative to disease onset and follow-up outcomes, will be needed to establish whether this signature has predictive value for disease progression or treatment response. As this study relies on a retrospective, publicly available dataset, we did not have access to the original biological samples for orthogonal validation (e.g., qPCR or Western blotting); independent replication in a separate cohort will be an important step for future work.

## 4. Materials and Methods

### 4.1. Datasets Selection

A systematic search was conducted across the ArrayExpress [37] and GEO [38] public databases to identify datasets meeting our predefined inclusion criteria. The search, performed on 4 April 2026, utilized the keywords “Blood” and “Multiple Sclerosis” to isolate relevant studies. To facilitate a robust transcriptomic analysis, the selection was restricted to datasets generated through microarray or RNA-seq technologies. Furthermore, to ensure statistical power and validity, only datasets incorporating substantial cohorts of HC, CIS, and RRMS samples were included. The availability of comprehensive metadata, specifically age and sex for each sample, was considered mandatory for inclusion in the final analysis. Among GEO entries, only one dataset included all three clinical groups of interest (HC, CIS, and RRMS); however, this dataset lacked sex and age annotation for the study participants, precluding adjustment for these covariates. Among ArrayExpress entries meeting the same clinical-group criterion, four studies were designed to evaluate the effect of specific pharmacological compounds rather than to characterize disease-stage-associated transcriptional differences, and were therefore not aligned with the aims of the present study; one further dataset lacked sex annotation, and two others included a substantially smaller number of samples per clinical group than E-MTAB-11415. E-MTAB-11415 was therefore selected as the dataset best meeting the required criteria: inclusion of HC, CIS, and RRMS groups without a pharmacological intervention design, complete age and sex annotation, and the largest per-group sample size among the available alternatives.

### 4.2. Datasets Information

As reported by the dataset publishers, peripheral blood samples were collected from all participants following the acquisition of informed consent. PBMCs were immediately isolated via discontinuous density gradient centrifugation using Lymphoprep (Nycomed, Oslo, Norway), and cell viability was assessed through Trypan Blue (Sigma-Aldrich, St. Louis, MO, USA) exclusion. Total RNA was subsequently extracted using Trireagent (Ambion, Austin, TX, USA) and stored at −80 °C. Prior to hybridization, RNA quantification and quality analyses were performed on a Bioanalyzer 2100 (Agilent, Santa Clara, CA, USA). Reverse transcription and biotinylated cRNA synthesis were conducted using the Illumina TotalPrep RNA Amplification Kit (Ambion) according to the manufacturer’s protocol. The samples were then hybridized to the arrays and scanned following standard Illumina hybridization and feature extraction procedures. Raw data processing, including background subtraction and Cubic Spline normalization, was performed using the Beadstudio software (version 3.2.2, Illumina Inc., San Diego, CA, USA).

### 4.3. Bioinformatics Analysis

Normalized probe intensities generated by BeadStudio were downloaded together with sample and probe annotation files. All the data were manipulated using R v.4.2.2 (R Core Team, Vienna, Austria). Expression values were log_2_-transformed and further normalized between arrays using quantile normalization (function “normalizeBetweenArrays”, limma v.3.54.2 [39], Bioconductor v. 3.16 [40]) to reduce residual technical variability prior to statistical modeling. A design matrix without intercept was built including clinical group (HC, CIS, RRMS) as factor and biological sex and age as covariates, following Equation (1):

(1) Expression = α1 · Condition + α2 · Gender + α3 · Age 

Models were fitted using lmFit, and group-specific contrasts (HC vs. CIS; CIS vs. RRMS) were extracted with functions “makeContrasts” and “contrasts.fit”, followed by empirical Bayes moderation of standard errors (eBayes). Raw *p*-values were adjusted for multiple testing using the Benjamini–Hochberg (BH) procedure; transcripts with an adjusted q-value < 0.05 were considered statistically significant DEGs in each comparison.

DEGs identified from the CIS vs. RRMS comparison were used for Over-Representation Analysis (ORA) of GO terms with the “clusterProfiler” v.4.6.2 [41]. The complete list of DEGs from the HC vs. CIS comparison, as well as the core gene set derived from the top-ranked GO categories in the CIS vs. RRMS comparison, were independently mapped onto the STRING protein–protein interaction database v.12.0 [42] (consulted on 1 June 2026) to explore functional connectivity among altered transcripts; the resulting networks were visualized and further analyzed using Cytoscape v.3.10.3 [43].

## 5. Conclusions

This study identifies a stage-dependent modulation of ribosomal and translational pathways in PBMCs from CIS and RRMS patients. CIS is characterized by downregulation of cytoplasmic and mitochondrial ribosomal genes, reduced expression of translation initiation and transcriptional components, and selective activation of stress-adaptive translational control. In contrast, RRMS is associated with reactivation of ribosome-related biological processes, suggesting increased biosynthetic and immunometabolic activity.

These findings support a translational stage-dependent model in which CIS is associated with translational restraint and stress adaptation, whereas established RRMS is associated with heightened ribosome biogenesis and protein synthesis capacity. The transcription–translation axis may therefore represent an underexplored layer of peripheral immune dysregulation in MS and a potential source of biomarkers for distinguishing disease stages and predicting progression from CIS to clinically defined MS.

## Data Availability

The microarray dataset analyzed during the current study is available in the ArrayExpress (BioStudies) repository under accession number E-MTAB-11415 (https://www.ebi.ac.uk/).

## Supplementary Materials

The following supporting information can be downloaded at: https://www.mdpi.com/article/10.3390/ijms27167340/s1.

## Abbreviations

The following abbreviations are used in this manuscript:

BH	Benjamini–Hochberg
BP	Biological Process
CC	Cellular Component
CIS	Clinically isolated syndrome
CNS	Central nervous system
CSF	Cerebrospinal fluid
DEGs	Differentially Expressed Genes
FC	Fold Change
GO	Gene Ontology
HC	Healthy controls
MF	Molecular Function
MRI	Magnetic Resonance Imaging
MS	Multiple sclerosis
OCB	Oligoclonal bands
ORA	Over-Representation Analysis
PBMCs	Peripheral blood mononuclear cells
PCA	Principal Component Analysis
RRMS	Relapsing-Remitting Multiple Sclerosis
UPS	Unfolded protein response
